# Impact of germline genetic variation on breast cancer prognosis: a systematic review and meta-analysis

**DOI:** 10.1186/s12885-026-15808-7

**Published:** 2026-03-04

**Authors:** Ariadna Gutiérrez-González, Eric Jonathan Maciel-Cruz, Nancy Reynoso-Noverón, Alejandro Mohar-Betancourt, Cynthia Villarreal-Garza, Lizbeth Grimaldo, Miguel Trujillo-Martínez, Liliana Gómez-Flores-Ramos

**Affiliations:** 1 School of Medicine and Health Sciences, Tecnologico de Monterrey, Av. Eugenio Garza Sada 2501 Sur, Nuevo León Monterrey, 2501 México; 2 Centro Integral de Oncología, Christus Muguerza, Hospital Betania, Av. 9 oriente 1814, Azcarate, Puebla, Puebla México; 3 Instituto Nacional de Cancerología, Av. San Fernando 22, Belisario Domínguez Secc 16, Tlalpan, Ciudad de México México; 4 Unidad de Investigación Biomédica en Cáncer, Instituto Nacional de Cancerología, Universidad Nacional Autónoma de México, Av. San Fernando 22, Belisario Domínguez Secc 16, Tlalpan, Ciudad de México México; 5 Breast Cancer Center, Hospital Zambrano Hellion TecSalud, Tecnologico de Monterrey, Av. Batallón de San Patricio 112, Real San Agustín, San Pedro Garza García México; 6 Secretaría de Ciencia, Humanidades, Tecnología e Innovación, Av. Insurgentes Sur 1582, Col. Crédito Constructor, Demarcación Territorial Benito Juárez, Ciudad de México México; 7 Centro de Investigación en Sistemas de Salud, Instituto Nacional de Salud Pública, Av. Universidad 655, Morelos Cuernavaca, México; 8 Medicina Familiar, Hospital General de Zona No. 7, Instituto Mexicano del Seguro Social (IMSS), Tulipanes 2, Centro, Cuautla, Morelos México; 9 Centro de Investigación en Salud Poblacional, Instituto Nacional de Salud Pública, Av. Universidad 655, Cuernavaca, México

**Keywords:** breast cancer, germline genetic variation, prognosis, survival, recurrence, meta-analysis

## Abstract

**Background:**

Breast cancer prognosis is the result of complex interactions between tumor biology, treatment, and host factors. While germline genetic variation is increasingly incorporated into breast cancer risk assessment and therapeutic decision-making, its role in determining prognosis remains incompletely defined. This systematic review and meta-analysis synthesizes available evidence on the association between germline genetic variants and breast cancer outcomes, with a focus on pathway-level effects.

**Methods:**

A comprehensive literature search was conducted in PubMed, Scopus, MEDLINE, Web of Science, and QInsight to identify studies published between January 2000 and June 2024. Published articles that evaluated associations between germline genetic variants and breast cancer prognosis were included. Eligible studies reported time-to-event outcomes, including disease-free survival (DFS), overall survival (OS), and other outcomes. Genes harboring prognostic variants were grouped into functional clusters using STRING. Primary analyses consisted of cluster-based multilevel random-effects meta-analyses, while global outcome-based meta-analyses were conducted as secondary, exploratory analyses. The protocol was registered in PROSPERO (CRD42022308746) and followed PRISMA guidelines.

**Results:**

Fifty-four studies encompassing 253,768 women were included. Most participants were of European ancestry, with marked underrepresentation of Hispanic (0.21%) and Black (0.25%) populations. Cluster-based meta-analyses identified six biologically coherent pathways associated with adverse prognosis. The ERBB2–PI3K resistance network showed the strongest association with OS (HR = 3.47; 95% CI: 1.54–7.78) and DFS (HR = 1.70; 95% CI: 1.02–1.82). Immune-related cytokine pathways were consistently associated with poorer OS (HR = 2.29; 95% CI: 1.04–5.04) and DFS (HR = 1.49; 95% CI: 1.20–1.85). Germline variation in xenobiotic metabolism genes was significantly associated with OS (HR = 1.65; 95% CI: 1.24–2.20), while DNA repair genes were strongly associated with breast cancer–specific mortality (HR = 3.51; 95% CI: 1.80–6.85). Exploratory global meta-analyses pooling all variants demonstrated overall directional associations with OS (HR = 2.30) and DFS (HR = 1.49), with substantial heterogeneity.

**Conclusions:**

This systematic review and meta-analysis show that germline genetic variation influences breast cancer prognosis in a pathway-specific manner. These findings highlight the importance of biologically informed analytic strategies and underscore the need for large, ethnically diverse studies to validate pathway-level germline prognostic markers and support their integration into personalized breast cancer management.

**Supplementary Information:**

The online version contains supplementary material available at 10.1186/s12885-026-15808-7.

## Background

Breast cancer (BC) is the most common cancer in females worldwide, and the incidence rates have kept increasing by 0.6% per year [1]. The prognosis for these women is widely heterogeneous due to the high diversity between tumors and individuals [2]. Prognosis relates to the risk of developing an outcome, such as recurrence or death, based on clinical and non-clinical characteristics [3]. Cancer recurrence is the reappearance of cancer after a period of absence of disease [4]. It can be classified into three main types based on the location of the recurrence: local, regional, and distant recurrence [5]. The latter occurs when cancer has spread to other organs or tissues far from the site of the primary tumor; this is called metastatic recurrence, and it has been reported that the majority of deaths from cancer of solid tumors are caused by metastasis [6]. Due to this, predicting the risk of breast cancer recurrence is of great importance in determining breast cancer prognosis. Understanding the risk of recurrence allows the physician to choose the best treatment and the optimal clinical management and surveillance for the patient.

Disease-free survival (DFS) is a clinical endpoint commonly used in oncology to measure the time after primary cancer treatment during which the patient survives without any signs or symptoms of the disease [7–10]. DFS is often used as a surrogate for overall survival (OS) in clinical trials [11]. OS is the period from diagnosis to death from any cause [12].

The prediction factors of recurrence for breast cancer used in clinical practice are age, clinical stage at diagnosis, histological grade, and molecular subtype [13]. In addition to these factors, genomic tools, known as genomic signatures, have been developed to calculate a patient’s recurrence risk score based on the expression of a set of somatic genes, such as Oncotype DX^®^ and MammaPrint^®^ [14, 15].

Another type of genomic prediction tool is risk scores created from germline variant information; this new approach has been under study in recent years. Germline variants have been used to determine an individual’s predisposition to develop cancer; however, it has also been reported that certain germline variants have high predictive value for recurrence [16–18].

Determining germline pathogenic/likely pathogenic (P/LP) mutations in BC already has significant implications in treating these patients. Identifying mutations in high-penetrance genes such as *BRCA1*,* BRCA2*,* PALB2*,* TP53*,* PTEN*, and *CDH1* can influence decisions on surgical and systemic treatments [19]. For example, the presence of germline *BRCA* (g*BRCA*) mutations can dictate the use of poly-ADP-ribose polymerase inhibitors (PARPi), such as Olaparib and Talazoparib, which have shown efficacy in improving outcomes in both early and metastatic settings [20, 21]. However, the relationship between these germline mutations and the patient’s prognosis is poorly defined. Some studies have reported worse clinical outcomes, whereas others have reported no difference in BC prognosis [22–24].

Recent research has also leveraged large-scale germline genetic datasets to explore broader biological pathways and causal mechanisms in breast cancer. For instance, Mendelian Randomization studies have evaluated associations between genetically regulated druggable targets and breast cancer risk [25] and examined potential causal links between breast cancer and structural brain changes [26]. Although these studies do not analyze prognostic outcomes, they illustrate the expanding scope of germline-based research in breast cancer and underscore the need for focused evaluations of germline variants specifically associated with survival endpoints.

Existing research has focused on individual germline variants in breast cancer prognosis. In contrast, this study aims to systematically synthesize germline variant–prognosis associations across multiple biological pathways and clinical outcomes using a pathway-based meta-analytic framework.

## Methods

### Study design and protocol registration

This study was conducted as a systematic review and meta-analysis to evaluate the association between germline genetic variants and breast cancer prognosis. The primary outcomes of interest included disease-free survival (DFS) and overall survival (OS), while secondary outcomes comprised breast cancer-specific survival (BCSS), progression-free survival (PFS), and metastasis-free survival (MFS), when reported. The study protocol was registered with the International Prospective Register of Systematic Reviews (PROSPERO; CRD42022308746) and adhered to the Preferred Reporting Items for Systematic Reviews and Meta-Analyses (PRISMA) guidelines [27].

### Search strategy

A thorough literature search was conducted across multiple databases, including PubMed, Scopus, MEDLINE, Web of Science, and QInsight. Studies published from January 2000 to June 2024 were included in the review. The search combined outcome-related terminology (e.g., prognosis, survival, recurrence) with two conceptual frameworks related to genetics: (1) germline variants and (2) germline polymorphisms. No language restrictions were applied, and searches were limited to studies involving adult women with breast cancer. Detailed Boolean search strategies, including all operators, subject headings, and database-specific filters, are provided in Additional File 1 (Tables S1–S3).

Studies were included in the analyses if they: (i) Investigated women with a confirmed diagnosis of breast cancer; (ii) Evaluated germline genetic variants, including single-nucleotide polymorphisms (SNPs) or pathogenic/likely pathogenic germline mutations; (iii) Reported at least one time-to-event outcome related to prognosis (such as DFS, OS, BCSS, PFS, or MFS; and iv) Provided an adjusted effect estimate. Exclusion criteria were applied to studies that did not assess germline genetic variation, failed to report survival or recurrence outcomes, were conference abstracts or review articles, lacked full-text availability, or presented duplicated data.

### Study selection and data extraction

Two reviewers independently assessed titles and abstracts for eligibility, followed by a full-text review of the selected articles. Any discrepancies were resolved through consultation with a third reviewer. The initial search yielded 576 records. After removing duplicates, 282 records were screened, and 68 full-text articles were assessed for eligibility. The article “Association of *BRCA1* K1183R polymorphism with survival in *BRCA1/2*-negative Chinese familial breast cancer” was excluded due to the unavailability of the full text [28].

**Fig. 1:**
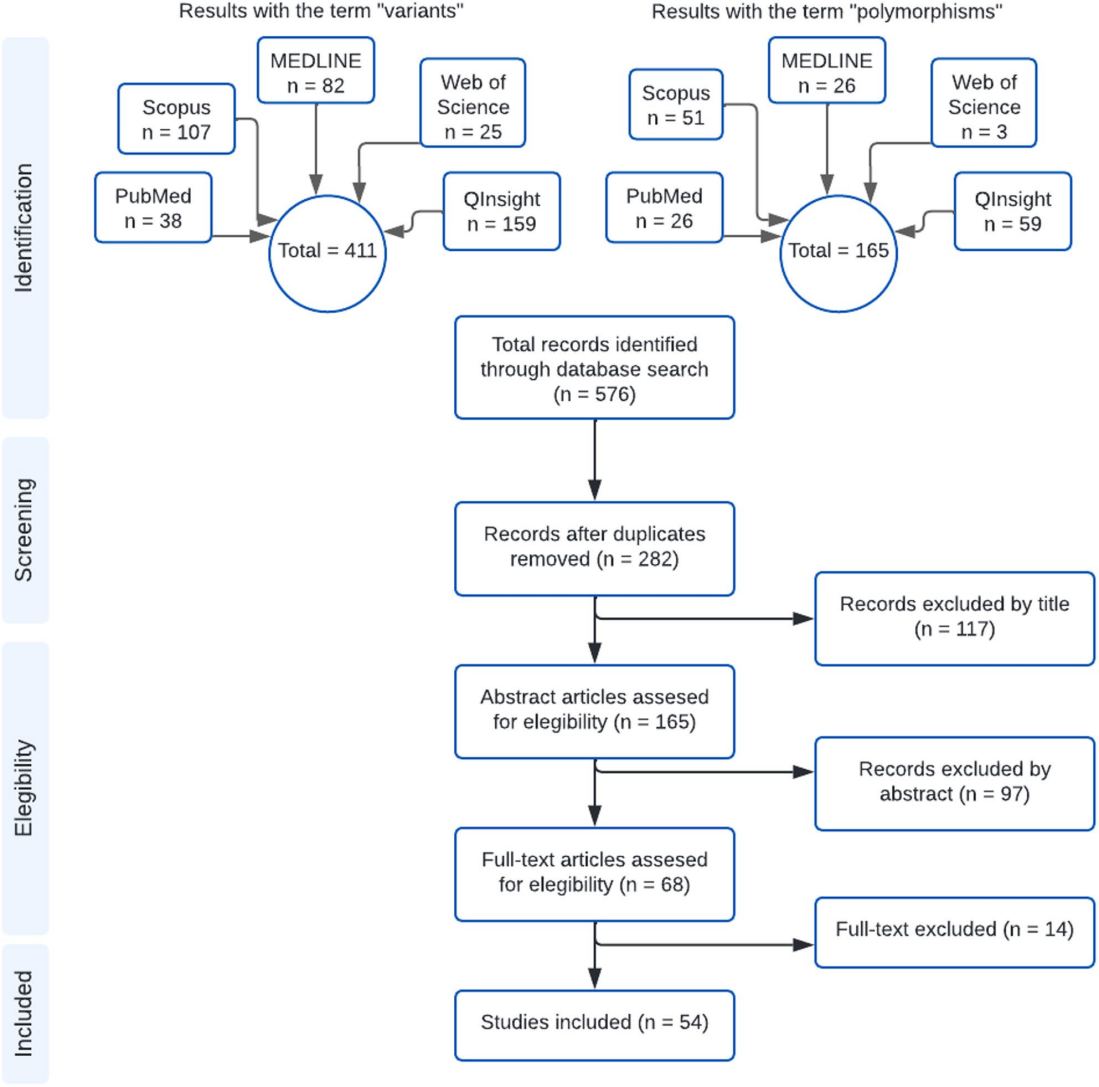
PRISMA flow diagram of the identification and study selection process

Fifty-six studies were included for thorough review and analysis. However, during data extraction, we found that no data about gene variants and measure of association was reported in the articles “Germline variants associated with leukocyte genes predict tumor recurrence in breast cancer patients” and “Effect of Adjuvant Paclitaxel and Carboplatin on Survival in Women With Triple-Negative Breast Cancer: A Phase 3 Randomized Clinical Trial” [29, 30]; therefore, we decided to exclude them. Ultimately, 54 studies were included in the final systematic review and meta-analysis, with the study selection process illustrated in the PRISMA flow diagram (Fig. 1).

Two data extractions were conducted independently by two reviewers using a standardized form (see Additional File 2). The extracted information included study design, population characteristics, ancestry, sample size, follow-up duration, tumor characteristics, reported treatments, and genetic variant information (such as gene name, variant identifier, allele or genotype, and allele frequency, when available). Additionally, we documented outcome definitions, adjusted effect estimates, and covariates included in multivariable models. In cases where multiple effect estimates were available for the same variant, the fully adjusted model was prioritized for extraction.

### Assessment of potential sample overlap

To reduce bias from non-independent samples, we assessed potential overlap among study populations by cross-referencing cohort names, consortium affiliations, institutions, recruitment periods, and author lists. This evaluation did not uncover any definitive evidence of overlapping datasets; however, we acknowledge that some residual overlap cannot be entirely ruled out and recognize it as a limitation.

### Functional clustering of genes

Most germline variants were reported in only a single study, making a variant-level meta-analysis infeasible. Therefore, the primary analytical approach involved grouping variants based on the functional and physical interactions of their respective genes. Gene mapping and clustering were conducted using the STRING database (2023 release) [31]. We used the full STRING network, including all interaction sources with a minimum required interaction score of 4.0 (medium confidence). The Markov Cluster Algorithm (MCL) was employed to identify biologically coherent gene clusters. These clusters were curated to ensure biological relevance, resulting in six functional gene clusters that represent key pathways related to breast cancer prognosis, along with a separate group for pathogenic *BRCA* mutations. The variants were grouped into six clusters. The final cluster composition was fixed prior to meta-analysis; Cluster 3 was modified, ADPQ and LEPR were removed, and TNFα was added to improve the biological coherence of the cluster, thereby strictly grouping genes involved in the immune cytokine signaling pathway.

### Statistical analysis

Primary analyses consisted of cluster-based multilevel meta-analyses, in which germline variants were grouped by biological function rather than pooled across unrelated genes. This approach was chosen to improve biological coherence, reduce conceptual heterogeneity, and enhance clinical interpretability. Exploratory global meta-analyses pooling all variants by outcome (DFS and OS) were conducted as secondary analyses to summarize the overall direction of association.

Adjusted hazard ratios (HRs) and 95% confidence intervals (CIs) were extracted and log-transformed. Standard errors were derived from the confidence intervals. Effect directions were harmonized so that HRs greater than one consistently indicated worse prognosis.

To account for statistical dependency arising from multiple estimates reported within the same study populations, multilevel random-effects meta-analyses were conducted using the rma.mv function in the metafor package (R version 4.5.2). Models included random effects at the study level and the estimate level. Restricted maximum likelihood (REML) estimation was used. This approach accounts for the hierarchical structure of the data by including random effects at the study level (between-study variance) and the estimate level (within-study variance).

### Cluster-based meta-analyses

Separate multilevel meta-analyses were conducted for each functional gene cluster and survival outcome when sufficient data were available. Pooled HRs and 95% CIs were estimated. Between-study heterogeneity was assessed using variance components and summarized using I². Statistical significance was defined as a two-sided p-value < 0.05.

### Global outcome-based meta-analyses

Secondary exploratory multilevel meta-analyses were conducted, pooling all eligible variants for DFS and OS. These analyses were intended to summarize overall directional trends and were not interpreted as precise prognostic estimates due to substantial biological and clinical heterogeneity.

## Results

A total of 54 studies met the inclusion criteria and were analyzed. Among the included studies, 37 were cohort studies, 14 retrospective studies, and 3 case–control studies, reflecting substantial methodological diversity. The majority of studies were conducted in Europe (*n* = 28), followed by Asia (*n* = 16) and North America (*n* = 8). Follow-up duration varied widely across studies, as did outcome definitions, covariate adjustment, and clinical characteristics, including tumor subtype and treatment exposure (Figure S1, Additional file 1).

Most studies (*n* = 52) reported at least one germline variant associated with breast cancer prognosis in univariate analyses, whereas 30 studies reported statistically significant associations in multivariable-adjusted models. Hazard ratios were the most frequently reported effect measure.

The total sample comprised 253,768 women, of whom the vast majority were White/Caucasian (89.40%). Asian participants accounted for 9.71%, while Hispanic/Latina (0.21%) and Black/African-American (0.25%) individuals were markedly underrepresented (Table 1). This imbalance was observed across both European and North American studies and highlights a substantial limitation in the generalizability of the findings, particularly for populations disproportionately affected by aggressive breast cancer subtypes and adverse outcomes.

**Table 1:** Ethnic distribution of the population included in the reviewed studies

Ethnic group	*n*	Percentage
White / Caucasian	226,884	89.40%
Asian	24,631	9.71%
Hispanic / Latina	543	0.21%
Black / African American	624	0.25%
No data / Other	1094	0.43%
Total	253,768	100%

The most common outcome was disease-free survival (DFS) (*n* = 34), and the most frequently used measure of association was the hazard ratio (HR) (*n* = 41). Most of the studies’ weaknesses were the lack of multivariate statistical analysis and the absence of statistical data, such as p-values and non-significant effect sizes.

HR is the most used measure in oncological research to analyze time-to-event. It quantifies the relationship between an independent variable and the outcome (survival) by computing the difference between the logarithms of the hazard functions. However, not all the studies included in this systematic review incorporated this risk estimate, and only a few adjusted for cofounders. Table 2 lists genes and variants associated with breast cancer prognosis, including adjusted HRs and statistical significance. Some studies grouped germline mutations rather than analyzing each one separately. Interestingly, all of them studied pathogenic mutations in the *BRCA* genes (as reported in Table 3).

**Table 2:** Descriptive table of variants that showed statistical significance in adjusted effect estimate by covariates [24, 32–55]. *ER positive, **PR positive, ***Lymph node metastasis

Author, Year	Population/Country	Sample size	Follow-up (mean years)	Confounders	Gene	ID	Allele/ Genotype	MAF/EAF	Outcome	HR/RRR	95% CI	*p*-value	Pathway
Zhu Qianqian, [36]	Trans-ethnic (European 70.50%, East Asian 11.32%, Hispanic 9.87% and African 8.31%)	3973	5	Age at diagnosis, body mass index, tumor grade, stage of disease, ER, PR, and HER2 status, hormonal therapy, chemotherapy, radiation therapy, surgery type, and all population stratification principal components.	*ARRDC3*	rs421379	T	0.069	OS	5.93	-	0.0145	Immune System
					*UACA*	rs720251	T	0.08	OS	2.79	-	4.19E-09	Programmed Cell Death - Apoptosis
						rs62019060	G	0.101	OS	3.01	-	1.27E-09	
Viktor Hlaváč, [37]	European	805	10	Tumor size and grade, lymph node metastasis, and estrogen receptor expression.	*CYP4 × 1*	rs17102977	G	0.09	DFS	1.69	1.01–2.85	0.048	Metabolism - Xenobiotics
					*CYP26B1*	rs62150087	G	0.09	DFS	0.54	0.33–0.89	0.016	Metabolism - Xenobiotics
Anna Morra, [38]	European	91,686	8.1	Age at diagnosis, ER status, PR status, HER2 status, tumor grade, and the use and type of systemic treatment.	*TBL1X*	rs5934618	G	0.08	OS	1.31	1.18–1.44	3.00E-07	Metabolism
					*LINC01487*,* STRIT1*	rs4679741	G	0.49	OS	1.2	1.13–1.28	1.10E-08	Metabolism - Estrogen biosynthesis
					*GRIP2*	rs1106333	A	0.06	OS	1.67	1.39-2	4.10E-08	Neuronal System
					*ARAP2*	rs78754389	A	0.07	OS	1.7	1.41–2.05	4.40E-08	Signal Transduction - RHO GTPase Cycle
Jolanta Pamuła-Piłat, [39]	Caucasian	305	5	Not listed.	*NR1/2*	rs3732359	AA	> 0.05	OS	1.82	1.24–2.8	0.003	Transciption factor - Xenobiotics Metabolism
					*SLC22A16*	rs7756222	CC	> 0.05	OS	1.58	1.05–2.36	0.027	Transport of small molecules
					*SLC22A16*	rs7756222	CC	> 0.05	PFS	1.57	1.07–2.32	0.021	
					*SLC22A16*	rs9487402	G	> 0.05	OS	1.72	1.14–2.59	0.009	
					*PGR*	rs1824125	GG	> 0.05	PFS	1.76	1.06–2.95	0.029	Metabolism of proteins
					*PGR*	rs12224560	CC	> 0.05	PFS	1.76	1.06–2.92	0.029	
					*DPYD*	rs291593	CC	> 0.05	DFS	5.89	1.29–26.88	0.022	Metabolism - Nucelotide catabolism
					*AKR1C3*	rs3209896	AG	> 0.05	DFS	5.49	1.2-25.05	0.028	Metabolism
Taru A Muranen, [24]	European	3008	15	Tumor grade, size, PR expression status, and lymph node involvement.	*DCAF1*	rs57025206	CC	0.023	OS	6.19	3.73–10.3	-	Cell cycle
Damien Coté, [40]	UK	157	10	Age, ER and PR status.	*ERBB3*	rs2229046	C	0.0495	DFS	2.79	1.91–4.95	1.51E-03	Gene expression
					*ERBB3*	rs773123	A	0.0665	DFS	2.67	1.02–7.03	0.05	
					*BARD1*	rs2070096	T	0.1903	DFS	3.27	1.16–9.17	0.05	Disease
					*ERBB2*	rs1136201	G	0.1556	DFS	2.67	1.05–6.78	0.05	Gene expression
Sinead Toomey, [41]	Irish	194	11.67	Age, tumor grade, ER state, LN state.	*EGFR*	rs2072454	C	0.416	OS	3.47	1.11–10.9	0.03	Signal Transduction
					*ERBB2*	rs1136201	G	0.247	DFS	2.36	1.02–5.5	0.04	
					*ERBB3*	rs2293347	C	0.09	OS	3.46	1.1–10.9	0.02	
					*EGFR*	rs1140475	T	0.125	DFS	6.51	1.98–21.36	0.01	
Rasa Ugenskienė, [42]	Lithuanian	100	5.83	Age at diagnosis, ER, PR, and HER2 receptors, tumor size, tumor grade and lymph node status.	*SIPA1*	rs3741378	T	NR	PFS	5.169	1.81–14.73	0.002	Immune System - Rap1 signalling
					*SIPA1*	rs3741378	T	NR	MFS	6.526	2.15–19.81	0.001	
Yu-Mian Jia, [43]	Chinese	1091	3.4	ER status, PR status, Her2 status, tumor size, clinical stage, lymphnode metastasis, chemotherapy and endocrine therapy status.	*CDH1*	rs7186053*	A	0.2965	DFS	0.29	0.12–0.67	0.0039	Signal Transduction - ESR mediated signalling
					*CDH1*	rs7186053**	A	0.2965	DFS	0.42	0.18-1	0.051	
					*CDH1*	rs7186053***	A	0.2965	DFS	0.35	0.13–0.95	0.0397	
					*CDH1*	rs7200690	T	0.2142	DFS	10.3	1.42–74.73	0.0211	
					*CDH1*	rs7198799	T	0.1517	DFS	10.91	1.13-105.34	0.0389	
					*CTNNB1*	rs4533622	A	0.2172	DFS	9.04	0.93–87.96	0.058	Signal Transduction
Dan-Na Chen, [44]	Chinese	715	6.12	Lymph node status, tumor size, chemotherapy, endocrine therapy, and ER, PR, and HER2 statuses.	*TLR3*	rs3775291	AA	NR	DFS	3.53	1.98–6.31	< 0.01	Immune System - TLR3 cascade
Petra Seibold, [45]	German	1408	6	Tumour size, nodal status, baseline metastases status, tumour grade, estrogen/progesterone receptor status, mode of detection, smoking status, menopausal hormone therapy as well as radiotherapy and chemotherapy.	*PARP2*	rs878156	G	0.07	BCSM	0.75	0.53–1.07	0.002	DNA repair
					*PARP2*	rs878156	G	0.07	BCSM	1.42	1.08–1.85	0.002	
Erika Korobeinikova, [32]	Lithuanian	100	5.83	Age group, tumor size, lymph node status, histological grade and intrinsic subtype.	*TNFα*	rs1800629	A	0.173	PFS	4.631	1.59–13.51	0.005	Signal Transduction - TNF Signalling
					*TNFα*	rs1800629	A	0.173	MFS	4.708	1.45–15.35	0.01	
					*TNFα*	rs1800629	A	0.173	OS	4.829	1.1-21.24	0.037	
C. Vulsteke, [46]	Belgium	991	5.2	Subtype, stage, White Blood Cell Count, and hemoglobin.	*CYP2C9*	rs1057910	C	NR	BCSS	30.4	6.1-151.5	> 0.001	Metabolism
					*ABCB1*	rs2032582	T	NR	BCSS	0.5	0.3–0.9	0.021	Drug ADME
					*CYP2C9*	rs1057910	C	NR	DFS	10.9	2.5–47.9	0.002	Metabolism
					*CYBA*	rs4673	T	NR	DFS	1.8	1.2–2.7	0.006	Immune System
					*UGT2B7*	rs3924194	G	NR	DFS	3.4	1.2–9.7	0.023	Drug ADME
Mala Pande, [47]	Trans-ethnic (72% were non-Hispanic white, 13% were African American, 11% were Hispanic and 3% were of unknown race/ethnicity).	1019	Not listed.	Race/ethnicity, age at diagnosis, tumor stage and treatment.	*ADIPOQ*	rs1063539	C	0.11	DFS	0.4	0.19–0.86	0.02	Metabolism
					*LEPR*	rs11585329	T	0.13	DFS	0.72	0.53–0.98	0.04	Metabolism of proteins
					*TSC1*	rs2519757	C	0.05	DFS	0.29	0.09–0.91	0.03	Autophagy
					*IGF1*	rs1520220	C	0.2	DFS	1.56	1.21-2	0.001	Growth and Development
					*PIK3CA*	rs2677760	C	0.045	DFS	1.43	1.07–1.92	0.03	Signal Transduction
Axel Muendlein, [48]	Austrian	161	4.2	TNM stage and age.	*IGF1*	rs2946834	A	NR	DFS	1.79	1.02–3.15	0.043	Growth and Development
Gudrun Absenger, [49]	Austrian	539	5.07	Menopausal status, stage, grading, receptor status, Her-2 neu status, and adjuvant therapy.	*VEGF-A*	rs3025039	T	NR	DFS	1.88	1.02–3.47	0.043	Angiogenesis
James L. Murray, [33]	Caucasian	997	18	Stage, age, and treatment.	*NFKB1*	rs230532	TT	NR	DFS	1.41	1.02–1.95	0.001	Immune System
	Caucasian				*IL-13*	rs1800925	GG	NR	DFS	1.47	1.04–2.07	0.034	
	African-American and Hispanic				*NFKB1*	rs3774932	GG	NR	DFS	2.52	0.91–2.89	0.02	
	African-American and Hispanic patients				*IL4R*	rs3024543	AA + AG	NR	DFS	1.57	0.93–2.64	0.03	
Peter A Fasching, [50]	Germany	20,073	6.4	Age at diagnosis and categorical variables for tumor size, lymph nodes status and grade.	*TOX3*	rs3803662	T	NR	BCSM	1.21	1.09–1.35	0.0002	DNA bending and unwinding
					*TOX3*	rs3803662	C	NR	BCSM	1.29	1.12–1.47	0.0003	
					*LSP1*	rs3817198	C	NR	BCSM	0.74	0.54-1	0.05	Immune System
Else Maae, [51]	Danish	116	5.1	Genotype, adjuvant chemotherapy, tumour size, axillary lymph node status and dichotomized histopathological tumour grade.	*VEGF-A*	rs2010963	C	0.37	DFS	2.57	1.05–6.3	0.04	Angiogenesis
Ke-Da Yu, [52]	Chinese	806	4.33	Lymph node status, tumor size, ER, HER2, and chemotherapy.	*NQO2*	rs2071002	C	NR	DFS	0.3	0.14–0.66	0.003	Metabolism - Functionalization
					*NQO2*	rs9501910	C	NR	DFS	1.59	1.07–2.37	0.023	
					*GSTM1*	Null/present	NA	NR	DFS	0.25	0.1–0.56	0.001	Drug ADME
Yee Soo Chae, [53]	Korean	240	4.45	Age, stage, histological grade, and ER, P53, and HER2 status.	*VARS2*	rs2074511	G	0.458	DFS	0.289	0.86–0.97	0.044	Metabolism of proteins - Aminoacylation
					*POLE*	rs5744857	G	0.315	OS	5.445	1.65-18	0.005	DNA repair
Verena Varadi, [54]	Swedish	783	4.7	ER and PR status, tumour size, lymph node metastasis, histological grade.	*REV3L*	rs11153292	C	NR	BCSS	3.67	1.56–8.62	0.003	DNA repair
				Size, grade, regional lymph node metastasis.	*REV3L*	rs462779	C	NR	BCSS	3.26	1.11–9.55	0.03	
Elizabeth M Azzato, [55]	European	3761	3	Stage, histopathologic grade, and ER status.	*OCA2*	rs4778137	G	0.3	OS	0.76	0.67–0.86	1.90E-05	Metabolism - Melanin biosynthesis
					*OCA2*	rs6626269	G	0.21	OS	1.35	1.19–1.52	2.20E-06	
					*OCA2*	rs4778137	G	0.3	OS	0.93	0.87–0.98	0.011	
					*OCA2*	rs6626269	G	0.21	OS	1.11	1.04–1.19	0.0025	
Gudrun Knechtel, [34]	Caucasian	216	10	Age at diagnosis, tumor size, lymph node status, clinical stage, histological grade, ER status, PR and treatment modalities.	*FAS*	rs2234767	A	NR	DFS	0.5	0.31–0.81	0.05	Programmed Cell Death
					*IL-10*	rs1800872	A	NR	OS	1.841	1.14–2.97	0.013	Immune System
Armin Gerger, [35]	Austrian	432	6.54	Age at diagnosis, tumor size, lymph node status, clinical stage, histological grade, ER status, PR status, and treatment modalities.	*IL-10*	rs1800872	A	NR	DFS	1.48	1.07–2.04	0.019	Immune System

Among the excluded studies, one should be considered. The study was conducted by Milanese et al. and presents results from the eTumorMetastasis algorithm applied to breast cancer patients. The eTumorMetastasis algorithm converts tumor functional mutations into network-based profiles to identify network operational gene (NOG) signatures [56]. These NOG signatures capture the transition point at which a tumor cell becomes likely to recur. The eTumorMetastasis algorithm was applied to exome sequencing data from 755 patients with estrogen receptor-positive (ER+) breast cancer. This algorithm identified gene signatures from germline variants that effectively distinguished between patients with and without recurrence in two independent cohorts (*n* = 200 and 295, *P* = 0.0014). These predictions outperformed the widely used Oncotype DX test for both high- and low-risk groups [29].

In addition to Milanese et al., other authors have developed algorithms to calculate scores that predict breast cancer prognosis, such as Maria-Escala Garcia et al., Ke-Da Yu et al., and Tuomas Heikkinen et al. [57, 58]. Table 4 describes these studies, as they showed statistical significance in multivariate analysis.

**Table 3:** Descriptive table of grouped mutations that showed statistical significance in adjusted effect estimate by covariates [18, 23]

Author, Year	Population / Country	Sample size	Follow-up (mean years)	Confounders	Gene	Type of variant	Outcome	HR	95% CI	*p*- value	Pathway
Matteo Lambertini, [23]	Europe, North America, Latin America, Israel	1236	7.9	Nodal status, grade, HER2, type of breast surgery, chemotherapy use, age, year of diagnosis, and country.	*BRCA1*	Pathogenic	DFS	0.76	0.6–0.96	-	DNA repair
Yong Alison Wang, [18]	Chinese	480	5	Tumor size > 2 cm, lymph node positivity, triple negative tumor type, young age of onset, mastectomy, adjuvant chemotherapy, adjuvant radiotherapy, and hormonal therapy.	*BRCA*	Pathogenic	DFS	3.04	1.4–6.06	0.05	
					*BRCA*	Pathogenic	DFS	2.7	1.2–6.06	0.016	
					*BRCA*	Pathogenic	DFS	2.86	1.11–7.35	0.029	
					*BRCA*	Pathogenic	OS	8.01	1.44–44.7	0.018	

**Table 4:** Descriptive table of scores developed to predict prognosis and showed statistical significance in adjusted effect estimate by covariates [52, 57, 58]

Author, Year	Population / Country	Sample size	Follow-up (mean years)	Confounders	Genes	Group	Outcome	HR	95% CI	*p*-value	Pathway
Maria Escala-Garcia, [57]	European	84,457	-	Not listed.	*ADRBK2*,* CCL16*,* CNR2*,* CXCR5*,* DNAJB4*,* F2R*,* GNA15*,* GNAT1*,* GRM4*,* GUCA1A*,* GUCA1B*,* GUCA2B*,* GUCY2D*,* HRH4*,* LTB4R*,* OPRK1*,* OPRM1*,* RGS9*, and *RGS9BP*	GRPM a1	OS	1.13	1.07–1.21	Mentioned as significant, but no p-value reported.	G-alpha signaling events
					*ADCY10*,* GNA11*,* PTGIR*, and *RGS3*	GRPM a2		1.15	1.08–1.22		
					*PER1*,* PER3*,* TIMELESS*, and *TIPIN*	GRPM b		1.28	1.19–1.37		Circadian clocks
					*CHCHD4*,* PDE9A*,* SLC36A1*, and *PHYHIPL*	GRPM c		1.2	1.12–1.28		Regulators of cell growth and angiogenesis
					*ARHGAP10*,* CCNT2*,* CDR2*,* HEXIM1*,* NEUROD2*,* PKN1*, and *ZFAND6*	GRPM d		1.21	1.16–1.28		Rho GTPases and apoptosis
Ke-Da Yu, [52]	Korean	240	4.45	Age, stage, histological grade, and ER, P53, and HER2 status.	*NQO2* and *GSTM1*	Combination of 3 SNPs: rs2071002, rs9501910 and null/present of GSTM1	DFS	0.25	0.1–0.63	0.005	Metabolism - Functionalization and Drug ADME
Tuomas Heikkinen, [58]	Finish	2204	6.92	Tumor size, nodal status, primary metastasis, estrogen receptor, progesterone receptor, HER2, p53, Ki67, grade.	*PTEN*	Promoter polymorphisms − 903GA, -975GC, and − 1026CA	BCSS	2.01	1.17–3.46	0.0119	Signal Transduction - Activation of AKT signaling
			3.92				MFS	1.79	1.03–3.11	0.0381	

### Functional clustering of prognostic germline variants

**Fig. 2:**
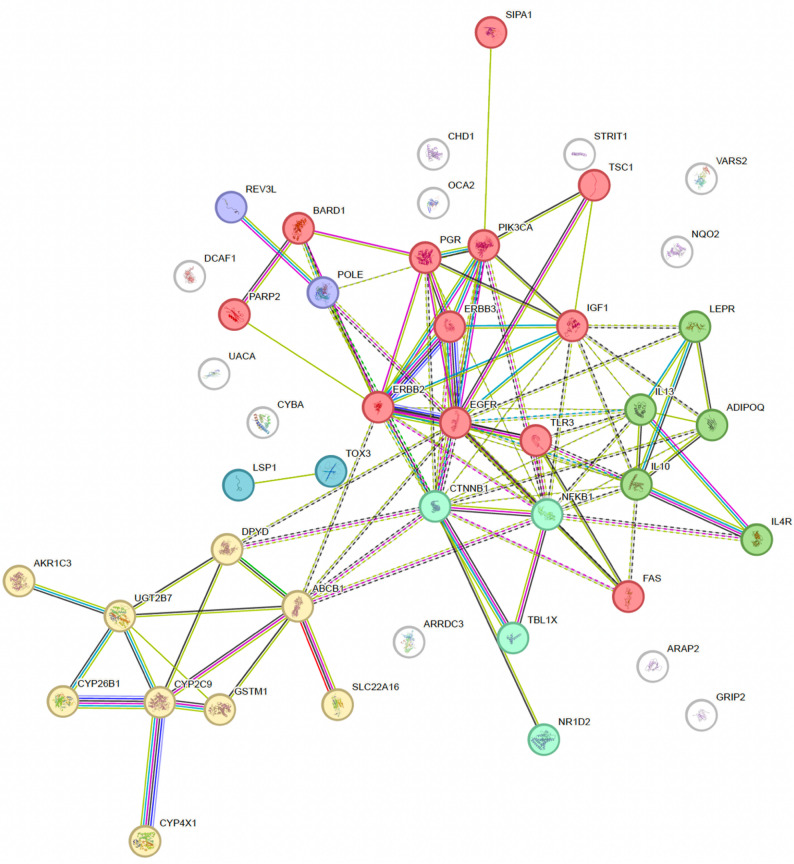
STRING mapping of the studied genes and STRING MCL clusters. Obtained by MCL clustering (finds natural clusters based on the stochastic flow) with an inflation parameter of 3 and dotted line as edges between clusters

Using STRING-based functional mapping, genes with prognostic germline variants were grouped into six biologically relevant clusters, representing key pathways involved in breast cancer biology (see Fig. 2; Table 5). These include growth factor signaling, drug metabolism, immune regulation, transcriptional control, cancer susceptibility loci, and DNA repair. A separate group consisting of pathogenic *BRCA* mutations was analyzed independently.

**Table 5:**
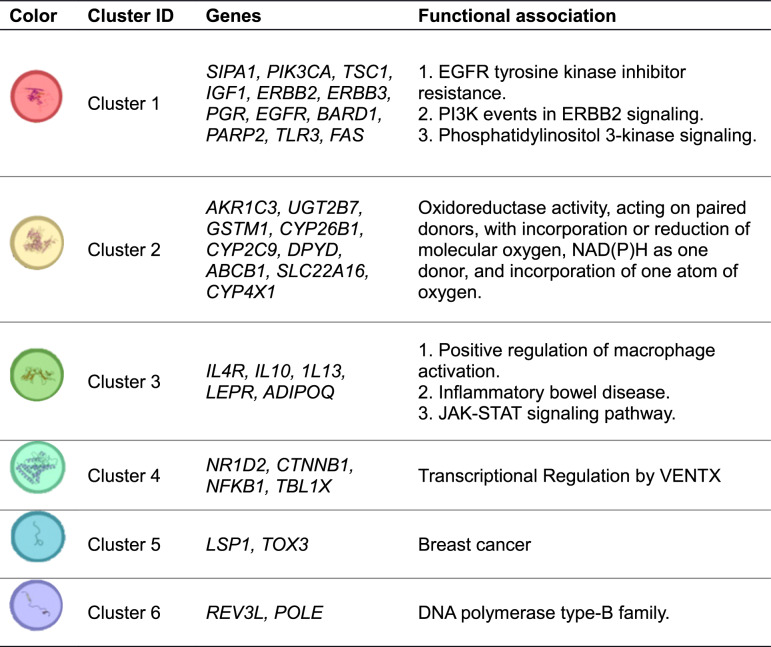
Descriptive table of STRING MCL clusters and their proteins

**Fig. 3:**
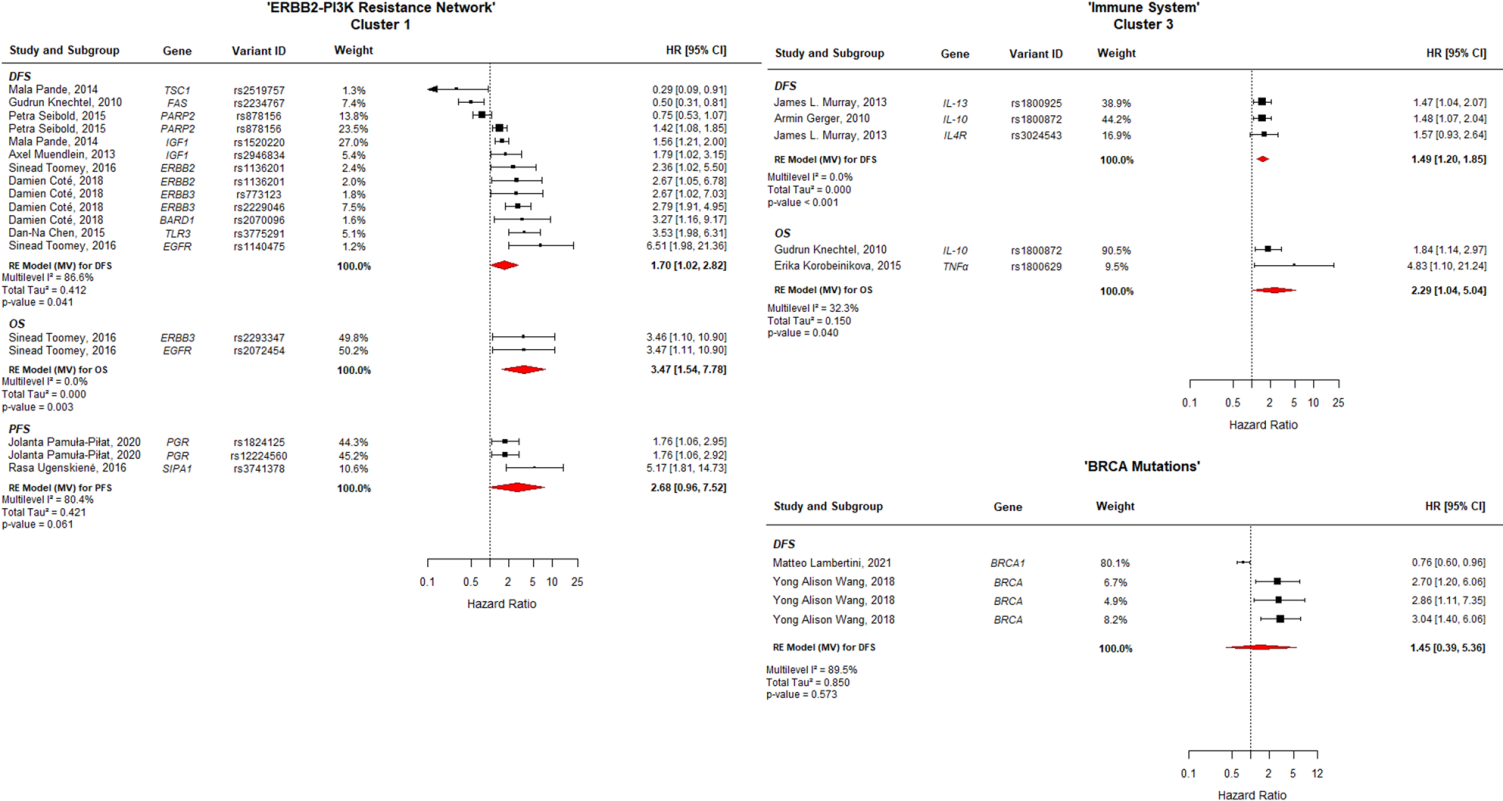
Forest plot of clusters 1, 3, and “BRCA Mutations”. The width of each estimate's square reflects the corresponding Standard Error (SE). DFS: Disease-Free Survival; OS: Overall Survival; PFS: Progression-Free Survival; CI: Confidence Interval

**Fig. 4:**
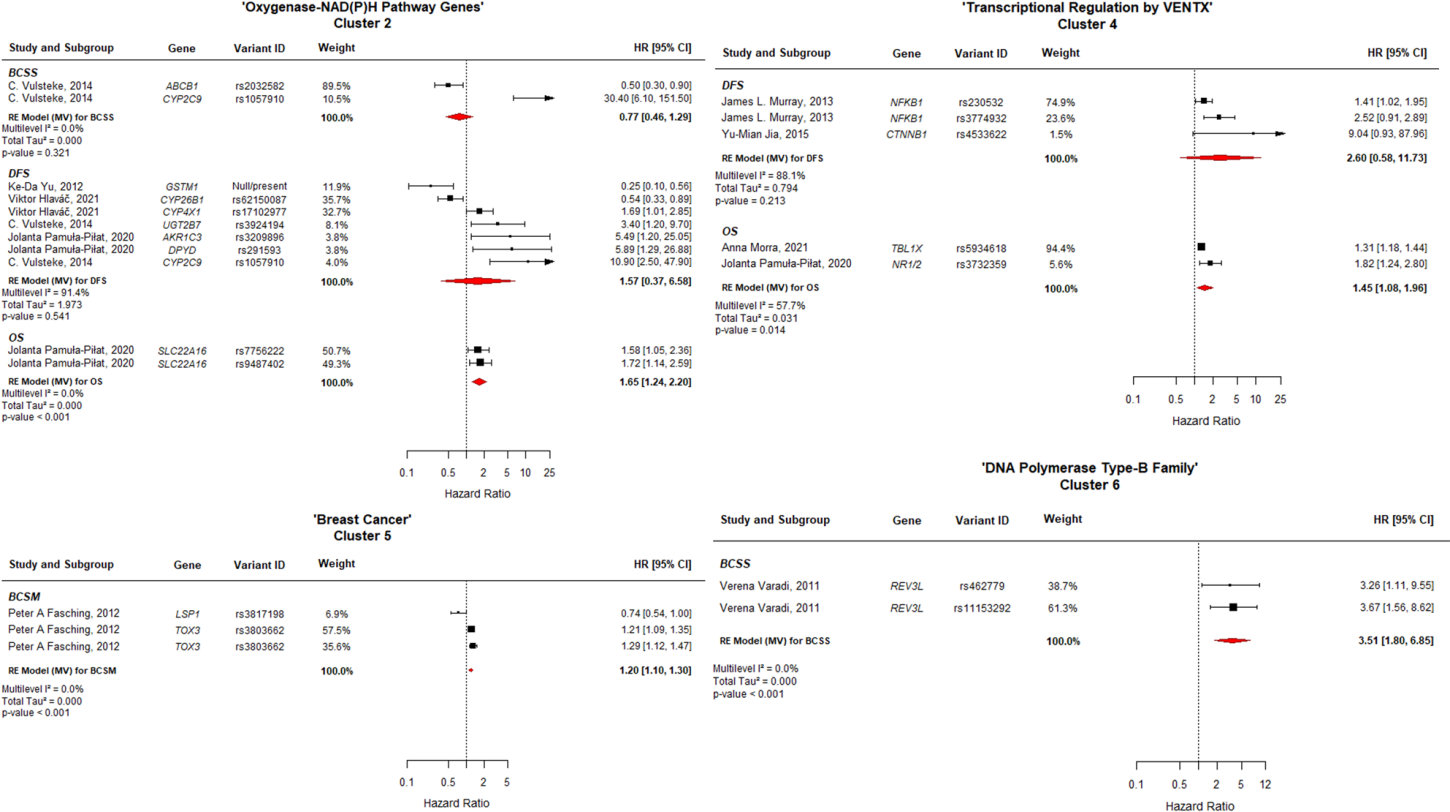
Forest plot of clusters 2, 4, 5, and 6. The width of each estimate's square reflects the corresponding Standard Error (SE). BCSS: Breast Cancer Specific Survival; DFS: Disease-Free Survival; OS: Overall Survival; BCSM: Breast Cancer Specific Mortality; CI: Confidence Interval

This clustering approach allowed for pathway-level synthesis of prognostic associations and served as the foundation for the main meta-analytic results (Figs. 3 and 4).

### Multilevel meta-analysis of prognostic outcomes

We performed a multilevel meta-analysis (RE Model, MV) for each gene cluster, stratified by clinical outcome.

### Cluster 1, “ERBB2-PI3K Resistance Network”

(grouping variants in *TSC1*,* FAS*,* PARP2*,* IGF1*,* ERBB2*,* ERBB3*,* BARD1*,* TLR3*,* EGFR*,* PGR*, and *SIPA1*), showed a significant association with poorer prognosis. The pooled HR for OS was 3.47 (95% CI: 1.54–7.78; *p* = 0.003), with no observed heterogeneity (I^2^ = 0.0%), representing the strongest association with OS among all analyzed clusters. Similarly, DFS was significantly compromised, with a pooled HR of 1.70 (95% CI: 1.02–1.82; *p* = 0.041) and high heterogeneity (I^2^ = 86.6%). The analysis for Progression-Free Survival (PFS) showed a trend towards increased risk (HR = 2.68; 95% CI: 0.98–7.52; *p* = 0.061; I^2^ = 80.4%) but did not reach statistical significance.

### Cluster 2: “Oxygenase-NAD(P)H Pathway Genes”

(including *ABCB1*,* CYP2C9*,* GSTM1*,* CYP26B1*,* CYP4 × 1*,* UGT2B7*,* AKR1C3*,* DPYD* and*SLC22A16*), showed differential effects across prognostic outcomes. A statistically significant negative impact on OS was observed, with a pooled HR of 1.65 (95% CI: 1.24–2.20; *p* < 0.001; I^2^ = 0.0%), suggesting that variations in drug metabolism and transport significantly influence mortality. In contrast, no significant associations were found for Breast Cancer-Specific Survival (BCSS) (HR = 0.77; 95% CI: 0.46–1.29; *p* = 0.321; I^2^ = 0.0%) or DFS (HR = 1.57; 95% CI: 0.37–6.58; *p* = 0.541; I^2^ = 91.4%).

### Cluster 3, labeled “Immune System”

(comprising *IL-13*,* IL-10*,* IL4R*, and *TNFα*), was consistently associated with poor prognosis across the analyzed outcomes (see Fig. 3). The multilevel model estimated a significant pooled HR for OS = 2.29 (95% CI: 1.04–5.04; *p* = 0.040; I^2^ = 32.3%). Similarly, DFS was significantly reduced in patients carrying these variants, with a pooled HR of 1.49 (95% CI: 1.20–1.85; *p* < 0.001) and no observed heterogeneity (I^2^ = 0.0%).

### Cluster 4 (Fig. 4), “Transcriptional regulation by VENTX”

 (grouping *NFKB1*,* CTNNB1*,* TBL1X*,* and NR1/2*), demonstrated a significant association with OS, showing a pooled HR of 1.45 (95% CI: 1.08–1.95; *p* = 0.014; I^2^ = 57.7%). However, the DFS analysis did not reach statistical significance (HR = 2.60; 95% CI: 0.58–11.73; *p* = 0.213) and exhibited considerable heterogeneity (I^2^ = 88.1%).

### Cluster 5: “Breast Cancer susceptibility loci”

(including *LSP1 and TOX3)*, demonstrated a statistically significant association with poorer outcomes for DFS (Fig. 4). The multilevel synthesis revealed a pooled HR of 1.20 (95% CI: 1.10–1.30; *p* < 0.001) with null heterogeneity (I^2^ = 0.0%), suggesting a consistent prognostic impact across studies.

### The analysis of Cluster 6 (REV3L, “DNA Polymerase Type-B Family”)

showed the strongest effect specifically for BCSS among the analyzed clusters (Fig. 4). The multilevel model for BCSS showed a pooled HR of 3.51 (95% CI: 1.80–6.85; *p* < 0.001) with no observed heterogeneity (I^2^ = 0.0%). This finding identifies germline variants in this translesion-synthesis polymerase as strong, independent predictors of cancer-specific mortality.

In the multilevel analysis restricted to DFS for *BRCA*-mutation carriers (Fig. 3), the pooled HR was 1.45 (95% CI: 0.39–5.36). This association was not statistically significant (*p* = 0.573) and showed high heterogeneity (I^2^ = 89.5%), suggesting that within this specific synthesis using the multilevel correction, *BRCA* status was not a consistent predictor of DFS.

### Global impact of germline variants on prognosis

**Fig. 5:**
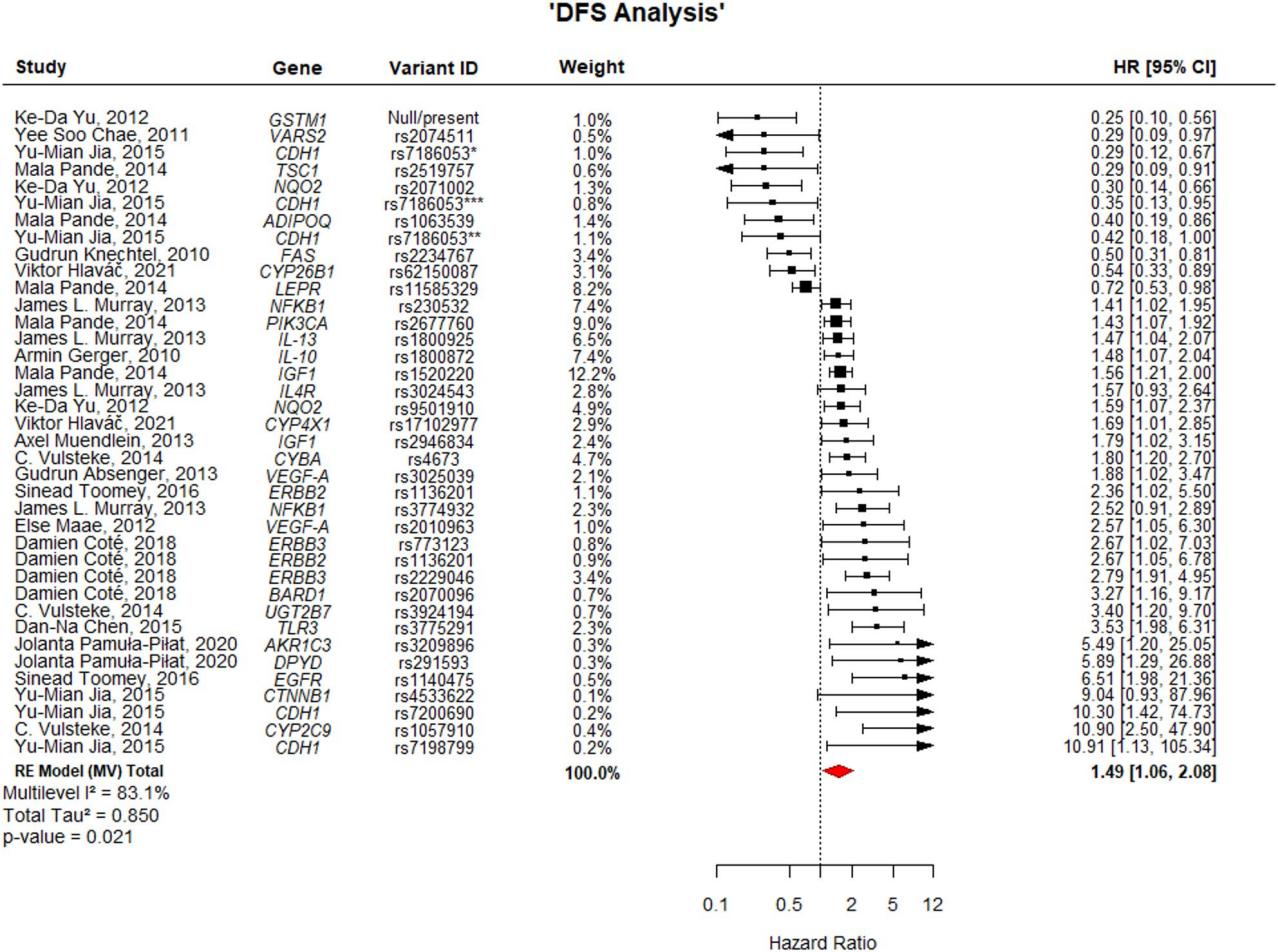
Forest plot of gene variants associated with DFS. The width of each estimate's square reflects the corresponding Standard Error (SE). DFS: Disease-Free Survival; CI: Confidence Interval

**Fig. 6:**
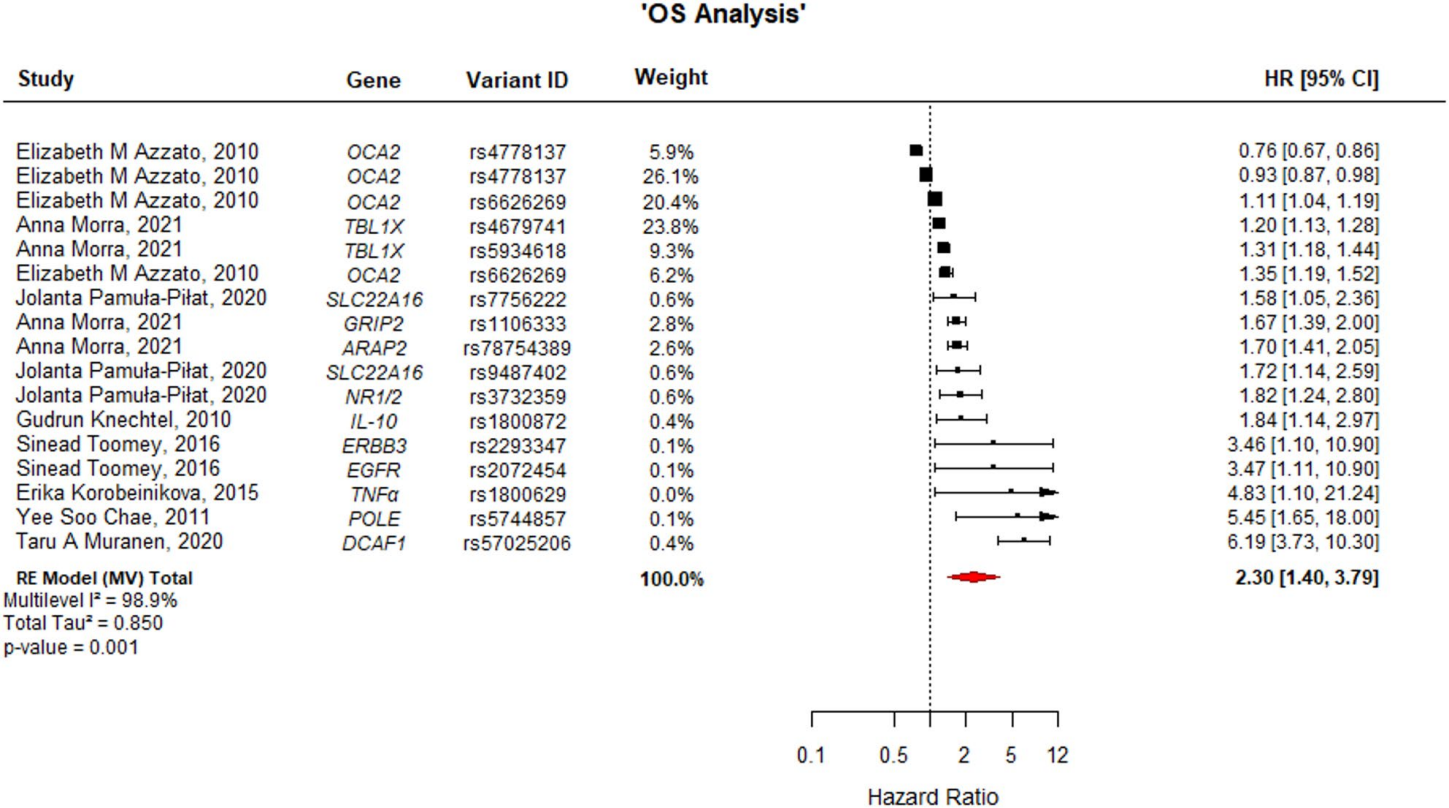
Forest plot of gene variants associated with OS. The width of each estimate's square reflects the corresponding Standard Error (SE). OS: Overall Survival; CI: Confidence Interval

Secondary multilevel meta-analyses pooling all eligible variants were conducted separately for DFS and OS to summarize overall directional trends. The analysis revealed a statistically significant association with adverse outcomes. For DFS (Fig. 5), the pooled HR was 1.49 (95% CI: 1.06–2.08; *p* = 0.021), with substantial heterogeneity (I^2^ = 83.1%). Furthermore, the association was even more pronounced for OS (Fig. 6), where the presence of the analyzed risk variants was associated with a more than two-fold increase in the risk of mortality (HR = 2.30; 95% CI: 1.40–3.79; *p* = 0.001), although heterogeneity remained high (I^2^ = 98.9%). These results support the hypothesis that, collectively, these germline genetic variations act as significant prognostic factors in breast cancer.

## Discussion

In this systematic review and meta-analysis, we compiled evidence from 54 studies involving over 250,000 patients to assess the prognostic significance of germline genetic variation in breast cancer. By focusing on biologically informed, cluster-based multilevel meta-analyses, we identified specific germline-mediated pathways linked to adverse clinical outcomes. Our results show that germline variants influence prognosis through coherent biological networks, especially those related to growth factor signaling, immune regulation, xenobiotic metabolism, and DNA repair, rather than functioning as isolated markers with uniform effects across clinical settings.

Overall, we identified 74 germline variants, five grouped pathogenic mutations, and seven groups of SNPs that were statistically significant in multivariate analyses associated with BC prognosis, indicating that germline DNA variations tend to be associated with BC prognosis across studies.

A key contribution of this work lies in the analytic framework adopted. Prior studies have largely focused on individual variants or single genes, often yielding inconsistent or context-dependent results. In contrast, our pathway-oriented approach accounts for the biological interdependence of genes and mitigates the limitations imposed by sparse replication of individual variants. This strategy revealed robust and clinically interpretable associations that were obscured in global analyses pooling heterogeneous variants. Importantly, the strongest and most consistent prognostic signals emerged from the cluster-based analyses, underscoring the value of aggregating germline variation at the pathway level for prognostic inference.

Among the identified clusters, the *ERBB2–PI3K* resistance network showed the strongest association with adverse outcomes, with a more than threefold increase in mortality risk and a significant elevation in recurrence risk. This pathway encompasses key regulators of cell proliferation, survival, and therapeutic resistance, including *EGFR*, *ERBB2*,* ERBB3*,* IGF1*, and downstream signaling components. The magnitude of the effect on OS, exceeding that observed for DFS, suggests that germline variation within this network may primarily influence disease lethality after recurrence, potentially by predisposing tumors to intrinsic or acquired resistance to systemic therapies. This interpretation aligns with extensive evidence implicating aberrant *ERBB2–PI3K* signaling in treatment resistance and aggressive tumor behavior. Biologically, this is highly consistent with the known role of the *ERBB/PI3K*/mTOR axis in driving tumor aggressiveness and mediating resistance to endocrine and anti-HER2 therapies. Extensive crosstalk between *IGF1R* and HER2 signaling pathways is a well-described mechanism of de novo resistance [59]. Germline variations in these pathways may constitutively activate downstream signaling or alter receptor sensitivity, potentially rendering standard treatments less effective.

In Cluster 2 “Xenobiotic Metabolism Enzymes and Transporters (XMETs)”, it is important to mention that the overall estimate HR for OS was 1.65 (95% CI: 1.24–2.20; *p* < 0.001) and this cluster includes some of the most frequently studied genes associated with BC prognosis, the cytochrome enzyme family (4 variants associated with BC prognosis). This family is primarily responsible for metabolizing most anticancer therapies. Studying the potential prognostic value of these genes could contribute to the development of new therapies, such as gene-directed enzyme prodrug therapy (GDEPT). Through GDEPT, *CYP* enzymes can be genetically modified to enhance the conversion of anticancer prodrugs into their active metabolites, thereby reducing prodrug dosage and minimizing chemotherapy side effects [60]. An example of this GDEPT is the use of oxazaphosphorines, such as cyclophosphamide (CPA) and ifosfamide (IFA), which are prodrugs activated by hydroxylation to produce cytotoxic metabolites like phosphoramide mustard. However, this metabolite is limited by its inability to cross cell membranes effectively when activated in the liver [61]. The P450 GDEPT strategy addresses this by delivering P450-expressing genes directly to tumor cells, enabling local activation and improved therapeutic effects, as demonstrated in a Phase 1 clinical trial using the MetXia-P450 vector with oral CPA. This approach has shown safety, consistent gene expression in cancer cells, and promising results, prompting further clinical trials [62].

We observed a consistent negative prognostic impact for clusters related to immune regulation. Cluster 3 (Immune System), comprising key cytokines (*IL-10*,* TNFa*,* IL-13*), was associated with a more than two-fold increase in mortality risk (OS HR 2.29). Germline variations in these genes can shift the tumor microenvironment towards an immunosuppressive state, facilitating immune evasion and metastasis [63]. Similarly, Cluster 4 (Transcriptional regulation by *VENTX*), which includes *NFKB1* and *CTNNB1*, was associated with poor OS (HR 1.45). *NFKB1* and *CTNNB1* are important regulators of inflammation and cell survival; constitutive activation of these pathways via germline variants may promote a chronic inflammatory milieu that supports tumor progression and stemness [64].

In contrast, pathogenic *BRCA1/2* mutations did not demonstrate a consistent association with disease-free survival in the multilevel analyses. While historically considered high-risk drivers, our multilevel meta-analysis for DFS did not reach statistical significance (HR 1.45; *p* = 0.57) and exhibited high heterogeneity (I^2^ = 89.5%). This contrasts with earlier single-estimate meta-analyses and likely reflects the complex clinical landscape of *BRCA* mutation carriers. In recent years, *BRCA* mutation carriers have benefited significantly from targeted therapies, such as platinum-based chemotherapy and PARP inhibitors, which can mitigate the negative prognostic impact of the mutation [20, 21]. In metastatic HER2-negative BC, g*BRCA* testing is recommended to prioritize platinum-based treatments, with trials demonstrating that PARPi improves progression-free survival and quality of life compared to chemotherapy [65–68]. Consequently, the prognostic value of *BRCA* status may vary substantially across treatment eras and regimens, which may explain the lack of a consistent signal across this heterogeneous pool of studies.

An interesting finding in this work is a new approach to creating scores based on germline genetic variants to predict BC prognosis, and how these scores can also highlight signaling pathways involved in BC recurrence, as Milanese et al. reported. The study found that the recurrent patients had a higher frequency of germline variants, particularly in genes related to T-cell function, antigen presentation, and cytokine interactions, which likely impair immune responses and promote a pro-tumorigenic environment [29]. This involvement of genes in immune system pathways was also observed in other articles in cluster 3 “Immune system”, such as *IL-10*,* TNFα*,* ILR-4*, and *IL-13* [32–35]. The overall estimate of cluster 3 for OS was 3.34 (95% CI 1.12–12.11) along with estimates for PFS and MFS higher than HR 4.5 [PFS HR 4.63 (95% CI 1.59–13.61); MFS HR 4.71 (95% CI 1.45–15.35)]. These findings open a new direction in the research of germline genetic variants and their association with BC prognosis.

The exploratory global meta-analyses pooling all germline variants by outcome revealed overall directional associations with disease-free and overall survival; however, these estimates were characterized by substantial heterogeneity and limited biological interpretability. These findings reinforce the notion that germline variants should not be treated as a homogeneous prognostic class. Rather, their effects are mediated through specific biological pathways and clinical contexts, which are better captured through structured, pathway-based analyses.

Our global analysis demonstrated that germline variants are independently associated with adverse outcomes. Specifically, the presence of risk variants was associated with a pooled HR of 1.49 for DFS and, notably, a more than two-fold increase in the risk of mortality (OS HR = 2.30). It is important to note the high heterogeneity observed (I^2^ > 80%), as it limits the generalizability of the average effect and highlights the need to consider differences when interpreting the results carefully. This is biologically plausible and expected, given the diversity of the included studies regarding patient ethnicity, tumor subtypes, treatment regimens, and the specific biological functions of the analyzed genes. However, the fact that the summary effect remains statistically significant despite this heterogeneity underscores the strong prognostic signal inherent in these germline factors.

Several limitations warrant consideration. First, the included studies exhibited considerable heterogeneity in design, population ancestry, outcome definitions, and covariate adjustment. Although multilevel modeling mitigated statistical dependence and variance inflation, residual heterogeneity remains unavoidable. Second, variant frequency data and treatment details were inconsistently reported, precluding refined stratified analyses by rare versus common variants or by specific therapeutic regimens. Third, despite the intentional search to include studies from diverse populations, this goal was not achieved because there were insufficient studies from non-white or Caucasian populations.

The diversity of study populations must be carefully considered, as Hispanic and Black individuals remain significantly underrepresented. As previously noted, most studies were conducted in Europe and Asia. Hispanic participants accounted for only 0.21% (543 out of 253,768 total participants), while Black participants represented just 0.25% (624 participants) in European and American studies. In stark contrast, White/Caucasian individuals comprised 89.57% (226,884 participants) of the total sample of included studies. These disparities limit the generalizability of the findings and highlight the urgent need for more inclusive clinical research that adequately represents these underserved populations. Finally, as with all observational genetic studies, causal inference cannot be established, and the identified associations may reflect complex interactions between inherited variation, tumor biology, and treatment exposure.

Despite these limitations, this study provides a comprehensive synthesis of the prognostic relevance of germline genetic variation in breast cancer and demonstrates the utility of pathway-based analytic strategies. Our findings suggest that germline information, when interpreted within biologically coherent frameworks, has the potential to complement established clinical and molecular prognostic factors. The strengths of this study include a comprehensive search strategy across five databases and the implementation of multilevel random-effects models. Unlike standard meta-analytic approaches, this method explicitly accounts for the hierarchical dependency of data arising from multiple polymorphisms or outcomes reported within the same study cohort. By correcting for this correlation, the associations reported herein are statistically robust and not artifacts of inflated sample sizes.

Our findings have potential implications for clinical practice as they suggest that identifying genetic variations linked to breast cancer prognosis could help to stratify the risk of recurrence and death to personalize treatment strategies. With the increase in genomic studies, germline testing for predictive variants will be incorporated into the management of breast cancer patients, and it must be accompanied by genetic counseling before and after testing. Genetic counseling enhances patient engagement, reduces patient distress, improves the accuracy of risk perception, and facilitates shared decision-making, which can improve clinical outcomes and quality of life [69–71]. Effective management would require a collaborative team comprising oncologists and geneticists in the clinical setting to address the new challenges associated with accurately interpreting genomic analyses.

New research should focus on validating identified germline genetic variants across diverse populations in large-scale studies and conducting longitudinal studies to better understand the temporal dynamics of these associations. Complete reports of the analyses, including covariate adjustments and data accessibility, are necessary for reproducibility studies. Integrative analyses that combine genetic, epigenetic, and environmental factors are necessary to create a comprehensive prognostic model.

Performing refined subgroup analyses, improving study design in underrepresented populations, leveraging advanced computational models, and standardizing variant interpretation could help researchers to better delineate the full clinical potential of germline variants in breast cancer. Ultimately, integrating germline genetic testing into routine Oncology care may enable more precise stratification of recurrence risk and improved outcomes for patients worldwide.

## Conclusions

This systematic review and meta-analysis emphasizes the importance of DNA germline variations in breast cancer prognosis. Our findings indicate that the prognostic impact of germline variation is pathway-specific, with certain biological networks exerting a disproportionately strong influence on disease lethality and survival after recurrence. While specific germline genetic variants have been identified as significant prognostic markers, further reproducible research is needed in diverse populations to fully understand their clinical relevance and integration into standard care practices. Understanding these genetic factors has the potential to lead to more personalized and effective strategies for managing breast cancer.

Future research should focus on validating these pathway-based associations in large, ethnically diverse cohorts with standardized outcome definitions and detailed treatment data. Integrative models that combine germline, somatic, and environmental factors will be essential to fully elucidate the role of inherited variation in breast cancer progression and to translate germline prognostic markers into clinically actionable tools. Ultimately, such efforts have the potential to refine risk stratification, inform personalized therapeutic strategies, and improve outcomes for patients with breast cancer.

## Supplementary Information

Supplementary Material 1. Additional file 1: Table S1. Search strings with the term “germline variants”; Table S2. Search strings with the term “germline polymorphisms”; Table S3. Complete search strings with Boolean operators for every database; and Figure S1. Graph representing the main methodology characteristics and results of the 54 articles included.

Supplementary Material 2. Additional file 2: Complete database of articles included.

## Data Availability

All data generated or analyzed during this study are included in this published article and can be consulted in Additional file 2.

## Abbreviations

CI: Confidence interval
BC: Breast Cancer
PRISMA: Preferred Reporting Items for Systematic Reviews and Meta-Analyses
DFS: Disease-free survival
OS: Overall survival
PFS: Progression Free Survival
MFS: Metastasis Free Survival
BCSS: Breast Cancer Specific Survival
BCSM: Breast Cancer Specific Metastasis
HR: Hazard ratio
NOG: Network operational gene
ER: Estrogen receptor
PR: Progesterone receptor
RRR: Relative Risk Ratio
LN: Lymph node
GDEPT: Gene-directed enzyme prodrug therapy
CPA: Cyclophosphamide
IFA: Iphosphamide
gBRCA: Germline BRCA
PARPi: PARP inhibitors
