# Supplementary material for: Impact of germline genetic variation on breast cancer prognosis: a systematic review and meta-analysis
Source: BMC Cancer. 2026 Mar 4;26:531. doi: 10.1186/s12885-026-15808-7 (PMC13122941; doi:10.1186/s12885-026-15808-7)
Supplement: Supplementary file 2 — Supplementary Material 2. Additional file 2: Complete database of articles included. [file 12885_2026_15808_MOESM2_ESM.docx]

**Search Strategy Details**

**Table S1. Search strings with the term “germline variants”.**

| Number of search | Search string |
| --- | --- |
| 1 | “Breast cancer recurrence” AND “germline variants” |
| 2 | “Breast cancer metastasis” AND “germline variants” |
| 3 | “Breast cancer prognosis” AND “germline variants” |
| 4 | “Breast cancer” AND “relapse free survival” AND “germline variants” |
| 5 | “Breast cancer” AND “recurrence free survival” AND “germline variants” |
| 6 | “Breast cancer” AND “disease free survival” AND “germline variants” |
| 7 | “Breast cancer” AND “distant metastasis free survival” AND “germline variants” |
| 8 | “Breast cancer” AND “progression free survival” AND “germline variants” |
| 9 | “Breast cancer” AND “event free survival” AND “germline variants” |
| 10 | “Breast cancer” AND “overall survival” AND “germline variants” |

**Table S2. Search strings with the term “germline polymorphisms”.**

| Number of search | Search string |
| --- | --- |
| 11 | “Breast cancer recurrence” AND “germline polymorphisms” |
| 12 | “Breast cancer metastasis” AND “germline polymorphisms” |
| 13 | “Breast cancer prognosis” AND “germline polymorphisms” |
| 14 | “Breast cancer” AND “relapse free survival” AND “germline polymorphisms” |
| 15 | “Breast cancer” AND “recurrence free survival” AND “germline polymorphisms” |
| 16 | “Breast cancer” AND “disease free survival” AND “germline polymorphisms” |
| 17 | “Breast cancer” AND “distant metastasis free survival” AND “germline polymorphisms” |
| 18 | “Breast cancer” AND “progression free survival” AND “germline polymorphisms” |
| 19 | “Breast cancer” AND “event free survival” AND “germline polymorphisms” |
| 20 | “Breast cancer” AND “overall survival” AND “germline polymorphisms” |

**Table S3. Complete search strings with Boolean operators for every database.**

| Database | Search string |
| --- | --- |
| PubMed | ("breast cancer"[tiab])  AND ("germline variants"[tiab] OR "germline polymorphisms"[tiab])  AND ("breast cancer recurrence"[tiab] OR "breast cancer metastasis"[tiab] OR "breast cancer prognosis"[tiab] OR "relapse free survival"[tiab] OR "recurrence free survival"[tiab] OR "disease-free survival"[tiab] OR "distant metastasis free survival"[tiab] OR "progression free survival"[tiab] OR "event free survival"[tiab] OR "overall survival"[tiab])  AND (2000:2024[pdat])  Filters: Humans, Female, Adult: 19+ years |
| Scopus | (TITLE-ABS-KEY("breast cancer"))  AND  (TITLE-ABS-KEY("germline variants" OR "germline polymorphisms"))  AND  (TITLE-ABS-KEY("breast cancer recurrence" OR "breast cancer metastasis" OR "breast cancer prognosis" OR "relapse free survival" OR "recurrence free survival" OR "disease-free survival" OR "distant metastasis free survival" OR "progression free survival" OR "event free survival" OR "overall survival"))  AND  (PUBYEAR > 1999 AND PUBYEAR < 2025) |
| MEDLINE | TS=("breast cancer")  AND  TS=("germline variants" OR "germline polymorphisms")  AND  TS=("breast cancer recurrence" OR "breast cancer metastasis" OR "breast cancer prognosis" OR "relapse free survival" OR "recurrence free survival" OR "disease-free survival" OR "distant metastasis free survival" OR "progression free survival" OR "event free survival" OR "overall survival")  Refined by:  Publication Years = 2000–2024  Document Types = Article |
| Web of Science | 1. breast cancer.ti,ab.  2. germline variants.ti,ab. OR germline polymorphisms.ti,ab.  3. (breast cancer recurrence OR breast cancer metastasis OR breast cancer prognosis OR relapse free survival OR recurrence free survival OR disease-free survival OR distant metastasis free survival OR progression free survival OR event free survival OR overall survival).ti,ab.  4. 1 AND 2 AND 3  5. limit 4 to humans  6. limit 5 to female  7. limit 6 to adult (19 plus years)  8. limit 7 to yr="2000 - 2024" |
| QInsight | ("breast cancer")  AND  ("germline variants" OR "germline polymorphisms")  AND  ("breast cancer recurrence" OR "breast cancer metastasis" OR "breast cancer prognosis" OR "relapse free survival" OR "recurrence free survival" OR "disease-free survival" OR "distant metastasis free survival" OR  "progression free survival" OR "event free survival" OR "overall survival")  AND year:2000-2022 |


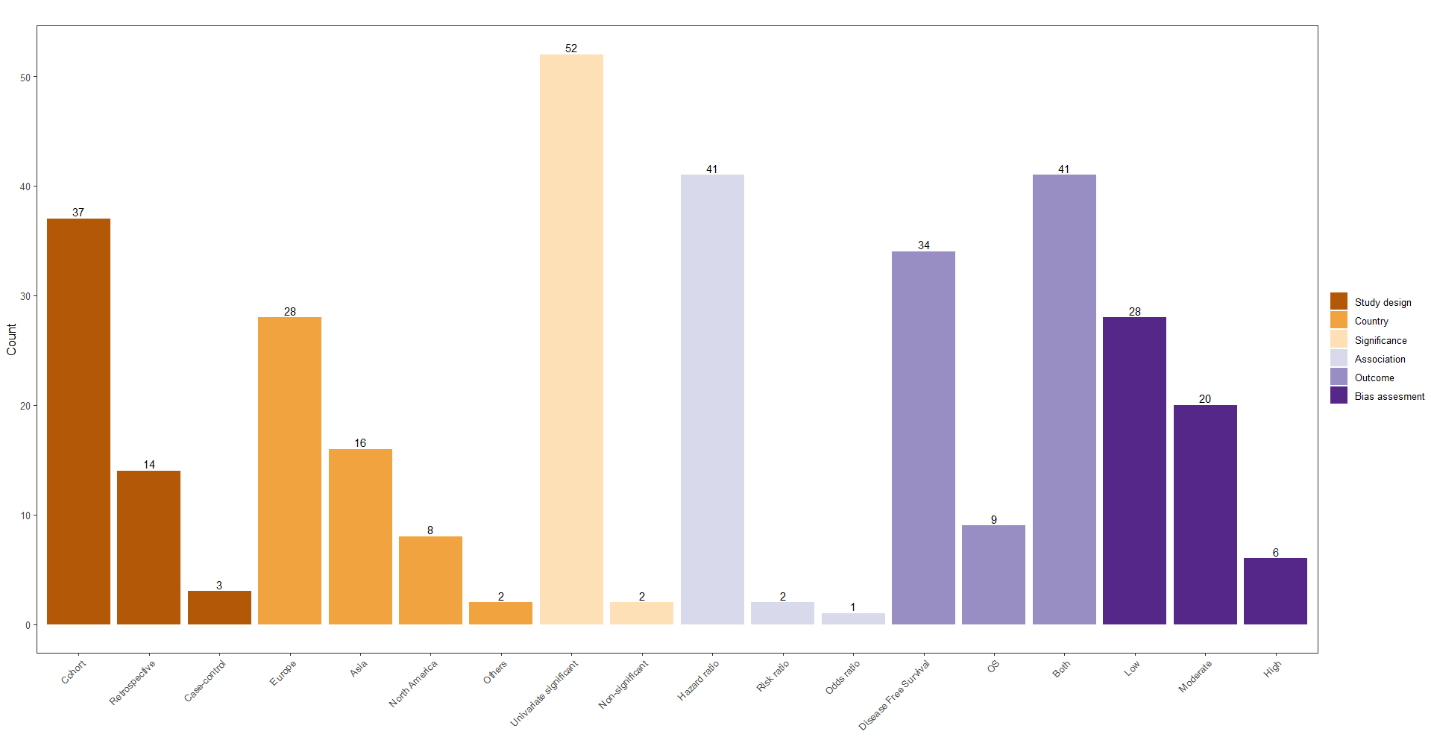


**Figure S1. Graph representing the main methodology characteristics and results of the 54 articles included.** Created with R Studio.
